# The effect of pre-operative MRI on the in-breast tumor recurrence rate of patients with breast cancer: a meta-analysis

**DOI:** 10.1007/s00423-025-03691-y

**Published:** 2025-04-04

**Authors:** Mahdieh Khoshzaban Banisi, Hani Ghadri, Behnaz Soltani, Amirali Farshid, Bahar Behnam, Amir Abbas Rhouholamini, Amirhossein Mohammadi, Seyedeh Fatemeh Hamzavi, Ashkan Azizi, Niloofar Deravi, Masoud Noroozi, Amin Magsudy, Sina Seyedipour, Shima Behzad, Yaser Khakpour

**Affiliations:** 1 Student Research Committee, School of Medicine, Shahid Beheshti University of Medical Sciences, Tehran, Iran; 2 School of Medicine, Iran University of Medical Sciences, Tehran, Iran; 3 Student Research Committee, Ahvaz Jondishapur University of Medical Sciences, Ahvaz, Iran; 4 Students Research Committee, Faculty of Medicine, Ardabil University of Medical Sciences, Ardabil, Iran; 5 Student Research Committee, School of Medicine, Tehran University of Medical Sciences, Tehran, Iran; 6 Department of Biomedical Engineering, Faculty of Engineering, University of Isfahan, Isfahan, Iran; 7 Tabriz Branch, Faculty of Medicine, Islamic Azad University, Tabriz, Iran, Islamic Republic of; 8 Jiroft University of Medical Sciences, Jiroft, Iran; 9 Independent Researcher, Islamic Azad University of Medical Sciences, Tehran, Iran; 10 Faculty of Medicine, Guilan University of Medical Sciences, Tehran, Iran

**Keywords:** Magnetic resonance imaging, Breast neoplasms, Preoperative evaluation, Recurrence, Meta-analysis

## Abstract

**Purpose:**

The impact of preoperative MRI on breast cancer recurrence and long-term outcomes remains undefined. Therefore, this study aims at determining the influence of preoperative MRI on in-breast tumor recurrence rates in cases of surgical treatment for breast cancer.

**Methods:**

A systematic review and meta-analysis were performed. Literature searches of PubMed, Scopus, and Google Scholar were conducted for studies up to February 2024. Two authors assessed the quality of the eligible studies and extracted their data.

**Results:**

The meta-analysis included 14 studies (2 RCTs, 12 cohort studies) with 12,889 patients with 5,451 undergoing preoperative MRI. Pooled hazard ratio for in-breast tumor recurrence was 0.95, using fixed effects and 0.94 using random effects models with 95% confidence intervals of 0.80–1.14 and 0.77–1.14, respectively. A trend towards lower recurrence rates in the MRI group was seen, but the reduction was not statistically significant.

**Conclusion:**

This meta-analysis found no significant reduction in in-breast tumor recurrence rates associated with preoperative MRI use in breast cancer patients, consistent with previous findings.

## Introduction

A positive surgical resection margin after breast-conserving surgery increases the risk of local disease recurrence and often requires additional surgery [1–3]. Preoperative breast Magnetic Resonance Imaging (MRI) is frequently used for local staging to assess tumor size and guide subsequent care. According to the guidelines published by the European Society of Breast Imaging, breast MRI can be used in three ways after breast cancer surgery: as a follow-up screening tool, to evaluate the possibility of local disease recurrence, and to identify any residual disease in the early postoperative phase [4].

As a result, the use of breast MRI has expanded globally in the hopes of improving surgical outcomes, reducing the risk of breast cancer recurrence, and improving survival rates [5, 6]. However, such results from preoperative MRI are still a source of discussion among clinicians with respect to its efficiency. Increased rates of false-positive results, re-excisions, or conversions from breast-conserving surgery to mastectomy have been attributed to MRI [7–11].

Preoperative imaging is used to assist with surgical planning, evaluate the need for neoadjuvant systemic therapy, and determine the extent of the disease in breast cancer patients [12–18]. According to current guidelines, the primary imaging modality should be bilateral mammography, with preoperative ultrasonography recommended if necessary [12, 18]. However, breast MRI is more expensive, requires intravenous contrast medium, and has moderate specificity compared to mammography, which increases the incidence of false positives [13–17, 19, 20].

After a diagnosis of breast cancer, contrast-enhanced breast MRIs are frequently performed before surgery to detect new lesions in the breast or obtain more details about the disease’s extent or distribution, which can aid in systemic therapy or surgery planning. Recent studies report sensitivity and specificity of over 90%, with some studies claiming rates as high as 97% [21, 22]. The Breast Imaging Reporting and Data System (BI-RADS) standard for MRI screening sensitivity is 85 to 90% [23]. While it is widely accepted that preoperative breast MRI can identify additional lesions, there is debate about whether detecting these lesions leads to improved patient outcomes [24].

A meta-analysis of four studies—three retrospective and one prospective—found no clear advantage of preoperative breast MRI. However, the reported retrospective studies vary in size, and most only document a small number of recurrent episodes [9, 25–29].

Given the ongoing debate on the use of preoperative MRI in the management of breast cancer, there arises a need for a proper evaluation of the impact on long-term outcomes, more so on in-breast tumor recurrence rates. This study attempts to answer the questions by carrying out a systematic literature review and meta-analysis of published articles. The following review synthesizes data collected from various individual studies in hopes of providing a more definitive answer to the question of whether preoperative MRI impacts in-breast tumor recurrence in patients undergoing surgery for breast cancer.

The primary purpose of this review is to assess how preoperative MRI influences in-breast tumor recurrence in patients with breast cancer. Other objectives include estimating the effects of preoperative MRI on surgical planning, the frequency of re-excisions, and overall survival rates. The objective of these analyses is to provide evidence-based information to healthcare providers in guiding the decision-making process for the incorporation of this modality in the management of breast cancer.

This meta-analysis extends prior publications by adding the latest studies and applying strict methodological approaches to ensure that our findings are valid and reliable. The results of this study have the potential to influence practice guidelines and contribute to the active debate on optimal use of preoperative MRI in the management of breast cancer.

## Materials and methods

### Study design

In this systematic review and meta-analysis, we aim to investigate the impact of preoperative MRI on in-breast tumor recurrence rates in patients diagnosed with breast cancer. Our methodology strictly adheres to the Preferred Reporting Items for Systematic Reviews and Meta-analyses (PRISMA) guidelines [30]. Additionally, the research protocol for this study has been registered with the Prospective Register of Systematic Reviews (PROSPERO).

### Search strategy

A comprehensive search of relevant articles was conducted until February 11, 2024, using databases such as PubMed, Scopus, and Web of Science. The search strategy consisted of three main groups of keywords, as outlined in Table 1. One group focused on Magnetic Resonance Imaging (MRI) terms, while the other two focused on breast cancer and recurrence, respectively. The groups were combined using the ‘AND’ operator, without any limitations on date, language, or publication type. The search strategy was modified to align with the specific query format of each database. To reduce the possibility of omitting appropriate papers, we thoroughly examined the bibliographies of related systematic reviews and incorporated accessible studies into our research. Both reviewers conducted all stages independently, resolving any disagreements through discussion.

**Table 1:** Search strategy for selected databases

Search engine	Search strategy	Additional filters	Total results
Pubmed	1,(MRI[Title/Abstract])OR(magne0cresonance imaging[Title/Abstract]),,,““"MRI"“[Title/Abstract]OR ““magne0cresonance imaging"“[Title/Abstract]”,“497,124”,04:51:01 2,breastcancer[Title/Abstract],,,““"breast cancer"“[Title/Abstract]”,“349,503”,04:51:58 3,(recurrence[Title/Abstract])OR (recurrent[Title/Abstract]),,,““"recurrence"“[Title/Abstract] OR""recurrent"“[Title/Abstract]”,“665,425”,04:52:28 4,(((MRI[Title/Abstract])OR(magne0cresonance imaging[Title/Abstract]))AND(breast cancer[Title/Abstract]))AND((recurrence[Title/Abstract]) OR(recurrent[Title/Abstract])),,,“(““MRI"“[Title/Abstract] OR""magne0cresonanceimaging"“[Title/Abstract])AND ““breastcancer"“[Title/Abstract]AND (““recurrence"“[Title/Abstract]OR ““recurrent"“[Title/Abstract])”,839,04:54:09	February 11, 2024	839
Scopus	(TITLE-ABS-KEY(mri) OR TITLE-ABS-KEY(“magnetic resonance imaging”)) AND TITLE-ABS-KEY(“breast cancer”) AND (TITLE-ABS-KEY(recurrence) OR TITLE-ABS-KEY(recurrent))	February 11, 2024	3972
Google Scholar	breast cancer recurrence MRI OR “magne0c resonance imaging” breast cancer MRI recurrence OR recurrent	February 11, 2024	183

### Inclusion and exclusion criteria inclusion criteria

To be included in this meta-analysis, studies met the following criteria:

1. Employed an observational methodology to eliminate potential interference from interventions.

2. Evaluated the association between preoperative MRI and in-breast tumor recurrence rate.

3. Included a population of patients diagnosed with breast cancer.

4. Clearly defined MRI and in-breast tumor.

Studies that employed experimental methodologies, were conducted on animal models, or focused on subjects with preexisting pathological conditions or outcomes other than breast cancer were excluded.

### Data extraction and quality assessment

Two reviewers were independently responsible for reviewing the title and abstract of each study to assess their eligibility for inclusion in this meta-analysis. Studies that did not meet the selection criteria were excluded from further consideration. The remaining studies underwent full-text screening and selected studies were included in the data extraction process. The following information was then extracted and categorized into four groups:

1. Study Characteristics: Information including authors, location, year of publication, and study design.

2. Patient-Specific Factors: Criteria determining the eligibility of breast cancer patients included in the study.

3. Study Design: The number of participants, sampling technique, and duration of sampling.

4. Outcomes: Assessment of the impact of pre-operative MRI on rates of in-breast tumor recurrence.

To ensure quality assessment, both reviewers utilized critical appraisal checklists specifically designed for cohort, case-control, and analytical cross-sectional studies, obtained from the Joanna Briggs Institute (JBI) (available at: https://jbi.global/critical-appraisal-tools). If there were disagreements, a third author was involved in the process.

### Statistical analysis

All statistical analyzes were performed using STATA 13.1 software (StataCorp LP, College Station, TX, UNITED STATES). Our results were presented as pooled hazard ratios (HRs) with a 95% confidence interval (CI) and visually represented using a forest plot. To assess heterogeneity between studies, we used the I^2^ statistic with a cutoff of 50% [31]. When notable heterogeneity was observed (I^2^ > 50%), we used the random effects model [32]. In addition, we performed a sensitivity analysis by systematically excluding one study at a time and repeating the meta-analysis to ensure the consistency of our results. Finally, we checked for possible publication bias using visual inspection of funnel plot symmetry and Egger regression analysis [33].

The statistical significance level was established at *P* < 0.05.

## Results

### Study selection and characteristics

**Fig. 1:**
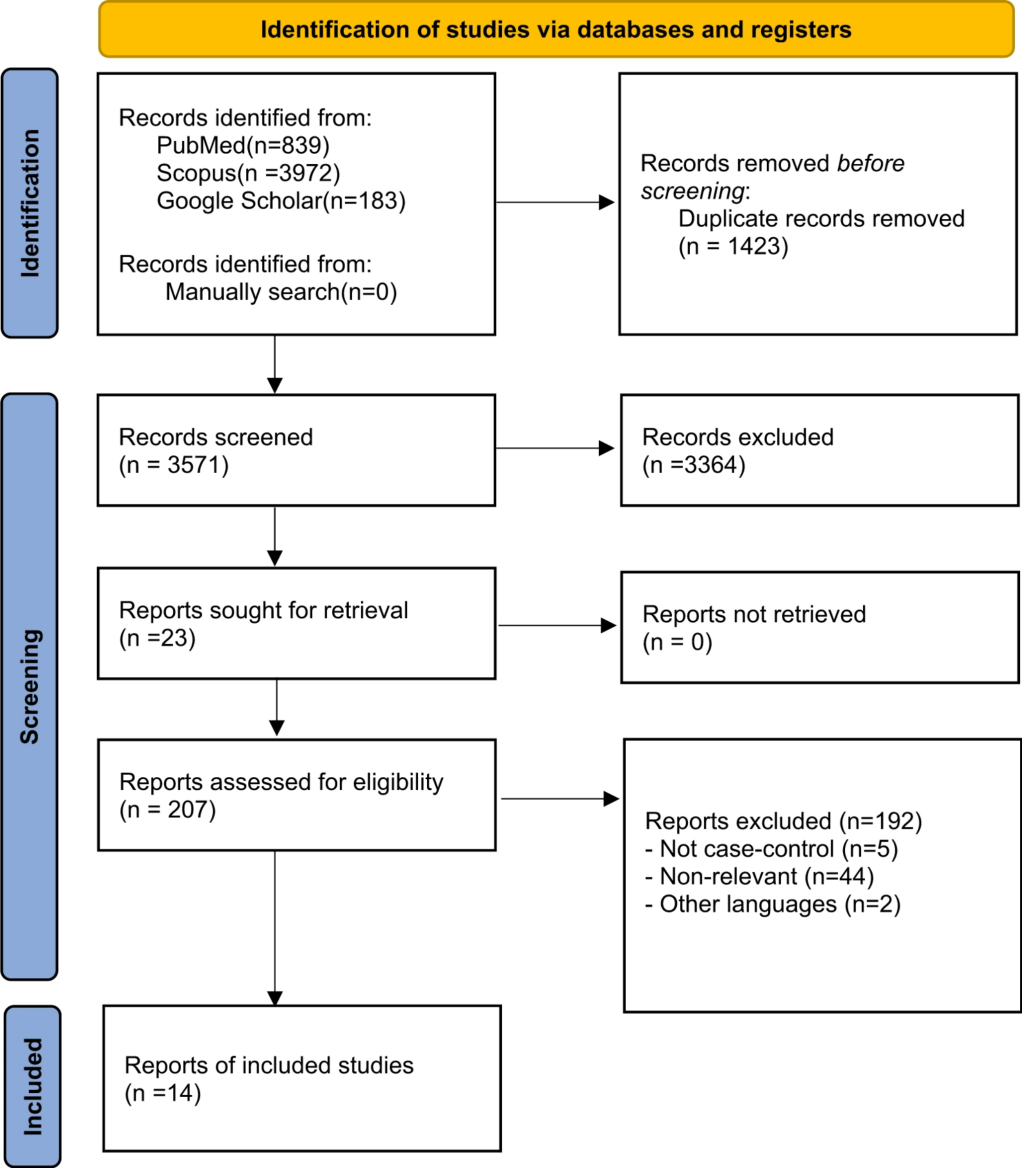
PRISMA flow diagram

A comprehensive literature search was conducted using three databases: PubMed, Scopus, and Google Scholar. The quality of the included studies was assessed. The initial search yielded 4,994 articles, which were reduced to 3,571 after removing duplicates. After applying inclusion and exclusion criteria and conducting primary and secondary screenings by two independent reviewers, 14 articles were selected for inclusion in the final analysis. A total population of 12,889 patients was reviewed. Of these, 5,451 had undergone preoperative MRI, while the remaining 7,438 did not receive preoperative MRI or had not assessed its impact on in-breast tumor recurrence. The PRISMA flowchart of the study selection process is shown in Fig. 1.

### Participant demographics

Of the studies, two are Randomized controlled trials (RCTs), while the rest are cohort studies. They were carried out in the United States of America [9, 34–37], the United Kingdom [38], Canada [29, 39], Sweden [5], and South Korea [27, 40–43], over the period from 2008 to 2023. Their ages ranged from 19 to 81 years, with the mean from 31.9 to 57.9. The follow-up for these studies went from 25.2 months to 165.6. In the MRI cohort, the number of participants ranged from 97 [27] to 816 [38]; in the non-MRI cohort, from 97 [27] to 1725 [35]. This variation was then carried further in-breast tumor recurrence rates between the MRI and non-MRI groups, with hazard ratios and their respective confidence intervals given, showing the relative risk and statistical significance (Table 2).

**Table 2:** Baseline characteristics of the included studies

Author/reference	Year	Country	Study design	Participants	Mean age	Intervention	Quality assessment score
Park et al. [43]	2023	South Korea	Retrospective cohort	708 women who were diagnosed with breast cancer	32 ± 3 years	Preoperative breast MRI	9/11
Gonzalez et al. [5]	2021	Sweden	Randomized multicentre study	440 patients with newly diagnosed breast cancer	46 years	Preoperative breast MRI vs. conventional imaging	10/13
Zeng et al. [47]	2020	United States	Retrospective cohort	512 women with a diagnosis of primary Stage 0-III breast cancer between January 2006 and December 2013, who under- went breast-conserving surgery as the initial treatment.	43.4 ± 5.0 years (MRI group), 43.6 ± 5.2 years (no-MRI group)	Preoperative breast MRI	9/11
Ha et al. [48]	2018	South Korea	Retrospective cohort	287 patients newly diag- nosed with ILC by biopsy or surgical excision between January 2005 and December 2012 and underwent subsequent treatment. 120 patients had undergone preoperative breast MR imaging (MR group) and 167 had not (no MR group).	49.8 years (range 31–82 years)	Preoperative breast MRI	11/11
Choi et al. [49]	2017	South Korea	Cohort	828 women with preoperative MRI and 1613 women without preoperative MRI	48.8 years (range 21–86 years)	Preoperative breast MRI	10/11
Hill et al. [34]	2017	United States	Cohort	1396 patients undergoing breast-conserving therapy at the institution between 2000 and 2010	60 ± 13 years (MRI group) 60 ± 11.6 years (no MRI group)	Preoperative breast MRI	9/11
Vapiwala et al. [36]	2017	United States	Retrospective cohort	755 women with ductal carcinoma in situ or early-stage invasive breast cancer underwent breast-conserving surgery followed by definitive breast radiotherapy.	Median age for MRI group: 53 years (range, 25–85 years); Median age for non-MRI group: 56 years (range, 27–89 years)	Breast-conserving surgery followed by definitive breast irradiation, with or without preoperative breast MRI	9/11
Gervais et al. [39]	2016	Canada	Retrospective cohort	470 patients with invasive breast cancer undergoing breast conservative surgery and radiation (27% underwent MRI and 73% did not)	56.8 years (range 25–92 years)	Preoperative breast MRI	9/11
Yi et al. [27]	2015	South Korea	Matched cohort study	742 unilateral patients and 194 bilateral patients	48.5 years (range 20–89 years)	Comparison of breast cancer disease-free survival outcomes between patients with and without preoperative MR imaging	10/11
Ko et al. [42]	2013	South Korea	Retrospective cohort	Total Participants: 1,271 patients who had undergone breast surgery. Breast-conservation surgery Attempted: 785 patients. Early-Stage Breast Cancer Treated with breast-conservation surgery and radiation therapy: 615 patients	46.6 years (range, 19–81 years)	MRI Group: 310 patients underwent preoperative breast MRI. Non-MRI Group: 475 patients did not undergo preoperative breast MRI.	8/11
Pilewskie et al. [35]	2014	United States	Retrospective cohort	2,321 women with ductal carcinoma in situ undergoing breast-conserving surgery from 1997 to 2010	57.9 years	Comparison of perioperative MRI vs. no MRI in women undergoing breast-conserving surgery for ductal carcinoma in situ, with and without radiotherapy	9/11
Turnbull et al. [38]	2010	United Kingdom	Randomized controlled trial	1623 women aged 18 years or older with biopsy-proven primary breast cancer who were scheduled for wide local excision after triple assessment.	57 years	Preoperative breast MRI	10/13
Hwang et al. [29]	2009	Canada	Prospective cohort	472 patients who underwent breast cancer surgery with final pathologic negative margins and received definitive adjuvant RT	MRI patients: 50 years (range 25–84 years); non-MRI patients: 58 years (range 30–92 years)	Preoperative breast MRI	9/11
Solin et al. [9]	2008	United States	Retrospective cohort	756 women with early stage invasive breast carcinoma or ductal carcinoma in situ underwent breast-conserving surgery including definitive breast irradiation	MRI group: 53 years (range, 25 to 85 years); non-MRI group: 56 years (range, 27 to 89 years)	Breast-conservation treatment including definitive breast irradiation	10/11

Abbreviations: MRI: magnetic resonance imaging

### Quality assessment

The quality assessment of the included studies revealed generally high standards, with most studies scoring above 80%. Specifically, Park et al. (2023) [43], Zeng et al. (2020) [37], Hill et al. (2017) [34], Vapiwala et al. (2017) [36], Gervais et al. (2017) [39], and Hwang et al. (2009) [29] each scored 9 out of 11. Gonzalez et al. (2021) [5] and Turnbull et al. (2010) [38] scored 10 out of 13, while Ha et al. (2019) [41] achieved a perfect score of 11 out of 11. Choi et al. (2017) [40], Yi et al. (2015) [27], and Solin et al. (2008) [9] each scored 10 out of 11. Ko et al. (2013) [42] scored 8 out of 11, and Pilewskie et al. (2014) [35] scored 9 out of 11. These scores indicate robust methodological quality across the studies, supporting the reliability of the meta-analysis findings.

### Meta-analysis of pre-operative MRI’s impact on in-breast tumor recurrence

**Fig. 2:**
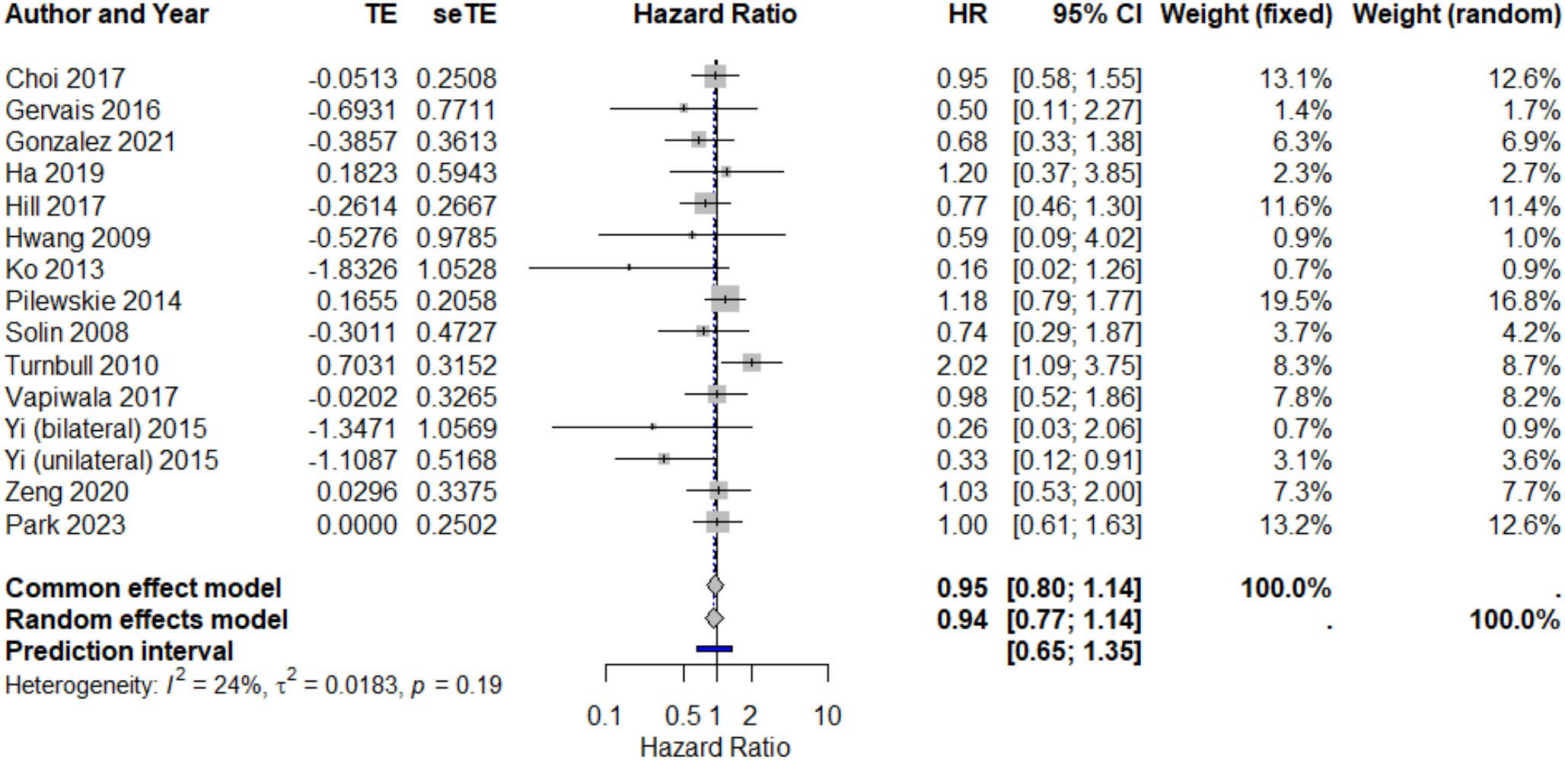
Forest plot of the effect of pre-operative MRI on the in-breast tumor recurrence rate of patients with breast cancer. Abbreviation: CI, confidence interval, HR: hazard ratio, TE: treatment effect, seTE: standard error of treatment effect

According to Fig. 2, we calculated the pooled hazard ratio for the defined outcome based on the information extracted from the 14 different studies included in this meta-analysis. Using the fixed effects model, the pooled HR was 0.95 (95% CI: 0.80 to 1.14), and using the random effects model, it was 0.94 (95% CI: 0.77 to 1.14). Both models suggest a trend toward risk reduction, but since 1 falls within the confidence intervals, this decrease is not statistically significant. Additionally, the prediction interval (1.00, 95% CI 0.65 to 1.35) indicates that the variability of treatment effects across studies is so high that future research might reveal either benefit or harm.

There was low to moderate heterogeneity between studies (I2 = 24%; *p* = 0.19), suggesting some between-study variance but not significantly. Individual study results were mixed, with some showing a beneficial effect (HR < 1) and others suggesting no benefit or even harm (HR > 1). Importantly, studies that used higher doses of the intervention, such as that by Pilewskie et al. (2014) [35], had a greater influence on the overall pooled estimate.

These results highlight the complexity and heterogeneity of treatment effects and emphasize the need for further research to identify a clearly effective treatment. While some studies have shown a relationship between preoperative MRI and breast tumor recurrence, our meta-analysis did not find a significant association, as illustrated in the Forest plot table (*p* < 0.05).

### Sensitivity analysis

**Fig. 3:**
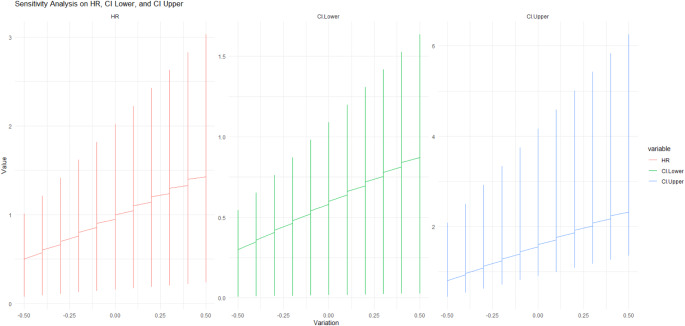
Sensitivity Analysis of the effect of pre-operative MRI on the in-breast tumor recurrence rate of patients with breast cancer. Abbreviation: CI, confidence interval, HR: hazard ratio, MRI: Magnetic resonance imaging

The graph of sensitivity analysis (Fig. 3) explores the HR and its related lower and upper bound of the 95% confidence interval (CI) by the increasing range of variation. In the left plot, the estimated HRs deviate around 1, with some estimates exceeding 3, as the variation increases. The range of lower bound monotonously increases from around 0 to over 4 in the figure of the central section, indicating the lower bound is increased with the increased uncertainty. The estimates of the hazard ratio and confidence intervals widen with increased variation; in the right panel, CIs sharply drop with values over 6. This confirms an increase in the uncertainty of the estimate with higher variation in the analysis and simultaneously underscores the resilience of our findings with different assumptions.

### Publication bias

**Fig. 4:**
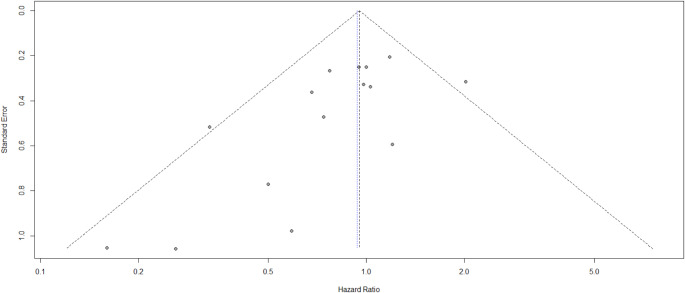
Funnel plot for publication bias assessment

The funnel plot (Fig. 4) is a graph correlating the standard error with the hazard ratio for the studies under review. A symmetrical configuration of this plot suggests that there is no notable publication bias present. The studies at the lower extremity of the plot represent small studies that are characterized by larger standard errors, and the larger studies, with reduced standard errors, aggregate around the mean hazard ratio at the upper extremity. This funnel plot is symmetrical in shape, thus indicating that publication bias is most likely not present among the articles. The sensitivity analysis identified no studies that should be excluded.

## Discussion

A meta-analysis of the available evidence for breast tumor recurrence was conducted to determine whether preoperative MRI reduces the rate of breast tumor recurrence. Our review of 14 studies involving 15,889 patients from various countries found that the use of preoperative MRI did not statistically significantly reduce recurrence rates. Aggregated hazard ratios from both fixed-effects and random-effects models were 0.95 (95% CI: 0.80–1.14) and 0.94 (95% CI: 0.77–1.14), respectively, indicating a small reduction in hazard. However, since the confidence intervals include 1, this reduction is not statistically significant.

Our results are in agreement with several others previously reported. In a cohort of 708 women, Park et al. (2023) [43] found an HR of 1.0 for overall recurrence (95% CI, 0.6–1.6; *P* = 0.99). In 512 women over 50 years old, no association of preoperative MRI use with improved clinical outcomes was found by Zeng et al. (2020) [37]. In another study, Gradishar et al. (2022) [44] reported no evidence of a variation in local recurrence rates between MRI and non-MRI cohorts. The authors emphasized that, although MRI has high sensitivity to detect additional tumor foci, this is not an automatic guarantee for an improved long-term outcome. Ha et al. [41], in a study involving 287 patients, did not find any significant relationship between preoperative MRI and the recurrence rates either. Such data support our contention that preoperative MRI may not significantly reduce recurrence rates.

Although preoperative MRI does not significantly lower recurrence rates, it could still be beneficial for certain patients. For example, individuals with multifocal or multicentric tumors, dense breast tissue, or those considering breast-conserving surgery may gain advantages in terms of surgical planning and margin evaluation. Further studies are needed to pinpoint which patient groups would benefit most from preoperative MRI, ensuring its targeted and effective use in clinical settings.

However, our results differ from those of several other studies. The single-center study by Gervais et al. (2017) [39] with 461 patients showed no statistically significant difference in the rates of ipsilateral breast tumor recurrence at 10 years between the MRI and no-MRI groups (1.6% versus 4.2%, respectively). These data support our conclusion that preoperative MRI may not significantly reduce recurrence rates.

This research unravels vital literature on the role of preoperative MRI in addressing breast cancer. Although MRI increases the capability of lesion detection and perceives a better illustration of the borders of tumors, according to our analysis, these better results cannot be shown to be connected with reduced recurrence rates. Our observation corresponds with that of Houssami et al. (2013) [45], who found that with preoperative MRI there was an increased use of surgery but no lower rate for re-excision or on the occurrence of a subsequent event.

However, our results differ from those of several other studies. Hill et al. (2017) [34] initially reported a significant reduction in recurrence associated with MRI (RR 0.6, 95% CI: 0.36–0.98, *p* = 0.04) in their univariate analysis; however, this association was not sustained in the multivariate analysis. They suggested that MRI could reduce local recurrence after breast conservation by identifying additional breast cancers or allowing for more complete tumor resection. Gonzalez et al. (2021) [5] reported a higher recurrence risk in the non-MRI group (HR 1.64, 95% CI: 1.00-2.67), contradicting our findings. This discrepancy might be attributed to their smaller sample size (440 patients) and focus on younger women (≤ 56 years).

The differences between our results and those reported by Hill et al. (2017) and Gonzalez et al. (2021) could stem from variations in study design, patient characteristics, or follow-up periods. For instance, Gonzalez et al. primarily studied younger women (≤ 56 years), who may have distinct tumor biology or imaging responses. Furthermore, differences in MRI protocols and surgical approaches among studies might also explain these inconsistencies.

The lack of any clear benefit in terms of reduced recurrence rates has associated cost-effectiveness considerations for routine preoperative MRI. According to Sardanelli et al. (2024) [46], the added sensitivity of MRI may result in overdiagnosis and overtreatment, thereby causally inducing excessive anxiety, increasing health care costs, but without proportionate benefits in terms of patient outcomes.

Since preoperative MRI does not clearly reduce recurrence rates, its cost-effectiveness is debatable. Health care providers should weigh the financial costs and risks of overtreatment before recommending routine MRI use in breast cancer care.

Strengths within our study are the large sample size, heterogeneous follow-up intervals, and patient demographics. However, we are fully aware that our study has a few limitations: not analyzing the subgroups based on patient characteristics, such as age or tumor size. This can possibly have an effect of study design and methods heterogeneity on our results. Our research has some limitations, such as not analyzing subgroups based on factors like age or tumor size. Future work should address these gaps by examining how preoperative MRI might benefit specific patient groups and exploring its influence on surgical results and quality of life.

Future studies should aim to identify subgroups of patients in whom preoperative MRI would have the most impact before its use is advocated for all surgical patients. Importantly, such future studies must also better define other possible advantages of MRI beyond recurrence rates, which may include, but are not limited to, surgical planning or patient outcomes. In this regard, clinicians should assess the preoperative MRI on a case-by-case basis, considering possible benefits against costs in terms of anxiety levels or risks of overtreatment, in view of the debates that are currently under way and of the conflicting results. In order to provide patients with the best possible care and therefore the best possible outcome in the treatment of breast cancer, the role that imaging technology can play must be continually reassessed as technology advances.

## Conclusion

In summary, although preoperative MRI does not markedly decrease breast tumor recurrence rates, it could still be useful for certain patients, particularly in surgical planning. Clinicians must balance its potential advantages against the risks of overtreatment and higher costs. Future studies should aim to identify which patients benefit most from MRI and investigate its effects on outcomes other than recurrence. Although MRI possibly presents benefits in improving lesion detection and possibly surgical planning, apparently, none of these is passed to effect on recurrence in the long term. Clinicians need to take a careful approach regarding the use of preoperative MRI according to individual benefit against cost and potential overtreatment risks. Future studies could determine subgroups of patients that might benefit the maximum from preoperative MRI studies and identify other potential benefits apart from recurrence. The ever-growing evolution in imaging technology warrants further evaluation in the context of race occurring in the management of breast cancer to offer optimized patient management and resource utilization.

## Data Availability

No datasets were generated or analysed during the current study.

## Glossary

BI-RADS: Breast Imaging Reporting and Data System.
PRISMA: Preferred Reporting Items for Systematic Reviews and Meta-analyses.
JBI: Joanna Briggs Institute.
RCT: Randomized controlled trial.
CI: Confidence Interval.
HR: Hazard Ratio.
MRI: Magnetic Resonance Imaging.
